# A positive-sense single-stranded RNA virus acquired a negative-sense open reading frame through recombination

**DOI:** 10.1371/journal.ppat.1013015

**Published:** 2025-04-08

**Authors:** Xuan Dong, Fan Zhang, Yiting Wang, Cixiu Li, Xuan Li, Chengyan Zhou, Guohao Wang, Zhongshuai Tian, Jing Gao, Hu Zhou, Liying Sui, Hans Nauwynck, Patrick Sorgeloos, Edward C. Holmes, Jie Huang, Weifeng Shi

**Affiliations:** 1 State Key Laboratory of Mariculture Biobreeding and Sustainable Goods, Yellow Sea Fisheries Research Institute, Chinese Academy of Fishery Sciences; Laboratory for Marine Fisheries Science and Food Production Processes, Qingdao Marine Science and Technology Center; Key Laboratory of Maricultural Organism Disease Control, Ministry of Agriculture and Rural Affairs, Qingdao Key Laboratory of Mariculture Epidemiology and Biosecurity, Qingdao, China; 2 Key Laboratory of Emerging Infectious Diseases in Universities of Shandong, Shandong First Medical University & Shandong Academy of Medical Sciences, Ji’nan, China; 3 Shanghai Institute of Virology, Shanghai Jiao Tong University School of Medicine, Shanghai, China; 4 Department of Analytical Chemistry, State Key Laboratory of Drug Research, Shanghai Institute of Materia Medica, Chinese Academy of Sciences, Shanghai, China; 5 Asian Regional Artemia Reference Center, Tianjin University of Science & Technology, Tianjin, China; 6 Laboratory of Virology, Faculty of Veterinary Medicine, Ghent University, Ghent, Belgium; 7 Laboratory of Aquaculture & Artemia Reference Center, Faculty of Bioscience Engineering, Ghent University, Ghent, Belgium; 8 School of Medical Sciences, The University of Sydney, Sydney, Australia; 9 Ruijin Hospital, Shanghai Jiao Tong University School of Medicine, Shanghai, China; University of California, Irvine, UNITED STATES OF AMERICA

## Abstract

Although positive- and negative-sense single-stranded RNA viruses are ubiquitous in nature, there is currently no evidence of recombination or reassortment between viruses with these two major forms of genome organization. Here, we describe the discovery of brine shrimp virga-like virus 1 (BSVV1), a novel positive-sense single-stranded RNA virus with a recombinant genome structure derived from two viral phyla with differing genome organizations. The genome of BSVV1 comprises three open reading frames (ORFs). ORF1 resembles the RNA-dependent RNA polymerase of Ips virga-like virus 1 (a positive-sense RNA virus), while ORF2, transcribed in the positive orientation, is related to the glycoprotein of Hubei bunya-like virus 10 and other negative-sense RNA viruses. The predicted ORF3 was unique to BSVV1 without known homologs identified. The presence of the three protein products was verified by mass spectrometry. Notably, our analysis also revealed that BSVV1 is geographically widespread and found in brine shrimp from at least eight countries on four continents. In addition, BSVV1 was successfully cultured and proliferated to high viral loads during brine shrimp development. In sum, we provide compelling evidence of an ancient recombination event between negative- and positive-sense single-stranded RNA viruses, enriching our understanding of the evolution of genome structures in RNA viruses.

## Author summary

The discovery of brine shrimp virga-like virus 1 (BSVV1) challenges the conventional view that positive- and negative-sense RNA viruses are evolutionarily independent. Through genomic and phylogenetic analysis, we show that BSVV1 has a global distribution and a unique genomic makeup, with evidence of a past recombination event between highly divergent positive- and negative-sense single-stranded RNA viruses. The identification of this novel virus, with protein products confirmed by mass spectrometry, expands our knowledge of RNA virus diversity and illuminates the mechanisms underlying genomic innovation. More broadly, the identification and characterization of BSVV1 provides valuable insights into the intricate processes shaping the genomic diversity and evolution of RNA viruses.

## Introduction

Positive-sense and negative-sense single-stranded RNAs represent two fundamental types of genome architecture in RNA viruses and are classified as different virus phyla [1,2]. Within this diverse genetic and phenotypic landscape, ambisense viruses, that possess genomes with regions of both positive- and negative-sense orientations, exhibit a unique genomic composition, with notable examples including tomato spotted wilt virus and melon severe mosaic virus from the order *Bunyavirales* [3–5]. As their positive-sense genomes must be transcribed into complementary antigenome segments prior to the synthesis of viral mRNA, ambisense viruses have been classified as negative-sense single-stranded RNA viruses [6].

The order *Martellivirales* comprises positive-sense single-stranded RNA viruses with unsegmented genomes that primarily infect plants. The *Martellivirales* is currently classified into seven families: *Bromoviridae*, *Closteroviridae*, *Endornaviridae*, *Kitaviridae*, *Mayoviridae*, *Togaviridae*, and *Virgaviridae* [7–12]. However, following large-scale metagenomic studies, many new members of the *Martellivirales* have now been found in animals [13] and commonly referred to as “virga-like viruses”.

The *Bunyavirales* is an order of segmented negative-sense RNA viruses that infect plants, insects, and vertebrates [14]. Most bunyaviruses are arthropod-borne and cause disease in a variety of animals, including humans [15]. The replication strategy of bunyaviruses is similar to other negative-stranded RNA viruses [16] although, as noted above, some bunyaviruses possess ambisense genome segments [5].

Although recombination events among DNA viral phyla and even between viral kingdoms have previously been described [17–20], there is currently no evidence for recombination or reassortment between positive-sense and negative-sense single-stranded RNA viruses.

Herein, we describe the discovery and characterization of an RNA virus from brine shrimp with the RNA-dependent RNA polymerase (RdRp) protein related to those of the virga-like viruses. However, it contains an ORF2 protein in a positive-sense orientation that was most closely related to that encoded by the M segment of several bunyaviruses. Hence, this represents an unusual recombination event between positive- and negative-sense single-stranded RNA viruses.

## Results

### Identification of the novel virus BSVV1 from brine shrimp

A total of 144 batches of brine shrimp cysts were collected from 21 countries and six continents between 1977 and 2019, and were pooled into 90 sequencing libraries. The samples encompassed all acknowledged brine shrimp species, namely: the sexually reproducing *Artemia franciscana*, *A. persimilis*, *A. salina*, *A. sinica*, *A. tibetiana*, *A. urmiana* and several strains of the parthenogenetic *Artemia* lineage [21]. Meta-transcriptomic sequencing and bioinformatics analyses identified one viral contig in 26 brine shrimp cyst libraries that exhibited amino acid similarity to virga-like viruses (order *Martellivirales*) in the RdRp. This was tentatively named brine shrimp virga-like virus 1 (BSVV1).

**Fig 1:**
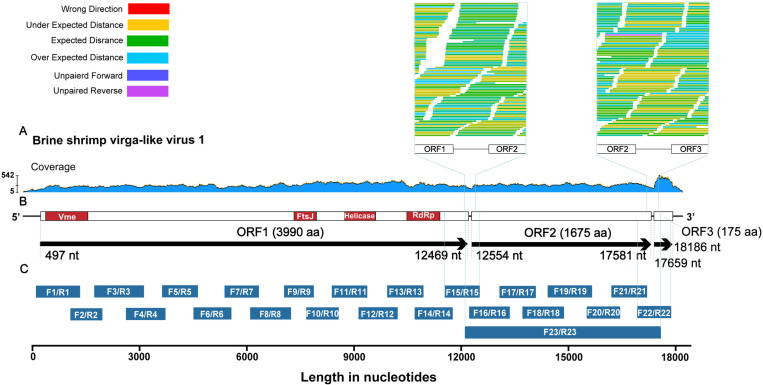
The genome sequence of BSVV1. (A) Coverage of the meta-transcriptomic sequencing reads mapped to the BSVV1 reference sequence (library KAZ-SL-20). Reads spanning both ORF1 and ORF2, as well as both ORF2 and ORF3, are highlighted. (B) Predicted structure of the full-length genome sequence of BSVV1 and the three predicted ORFs, as well as functional motifs. (C) The overlapping primers designed to amplify the BSVV1 genomic fragments using RT-PCR for Sanger sequencing, where F23/R23 is a primer that spans the three regions of ORF1, ORF2, and ORF3.

The relative abundance of BSVV1 ranged from 1.63 to 216.81 reads per kilobase million mapped reads (RPKM) across the 26 sequencing libraries (S1 Table). The BSVV1-positive samples were collected from 1991 to 2019 in eight countries from four continents, comprising China (n=12), Russia (n=5), and Kazakhstan (n=2) in Asia, Libya (n=3), Algeria (n=1), and Tunisia (n=1) in Africa, Australia (n=1) in Oceania, and Italy (n=1) in Europe (S1 Table). These samples belonged to three species – *A. salina*, *A. sinica*, *A. tibetiana*, and the parthenogenetic *Artemia* lineage (S1 Table). To confirm the presence of BSVV1, we performed read mapping for the assembled contig from library KAZ-SL-20, which comprised samples collected from Kazakhstan and in which no other virus sequences were found. A total of 27248 non-repetitive reads were mapped to BSVV1 with a mean depth of 217.5±69.8, yielding a consensus of 18125 nt in length. Importantly, tens of sequence reads spanned both ORF1 and ORF2, as well as ORF2 and ORF3 (Fig 1A and 1B).

### Annotation of the complete genome sequence of BSVV1

To validate the integrity of our genome assembly, we designed overlapping primers (S2 Table) and amplified the virus genome from samples KAZ-SL-20 (F1/R1-F22/R22) and 1816 (F23/R23) for Sanger sequencing. Notably, primers F15/R15 and F22/R22 were designed to amplify the gene regions spanning both ORF1 and ORF2, and both ORF2 and ORF3, respectively, while F23/R23 spanned ORF1, ORF2, and ORF3 (Fig 1C). The genome sequences obtained from Sanger and meta-transcriptomic sequencing were identical. Further determination of the termini of the viral genome was achieved through 5’ and 3’ rapid amplification of cDNA ends (RACE) experiments using sample KAZ-SL-20, and the full-length genome sequence of BSVV1 was determined as 18463 nt in length.

**Fig 2:**
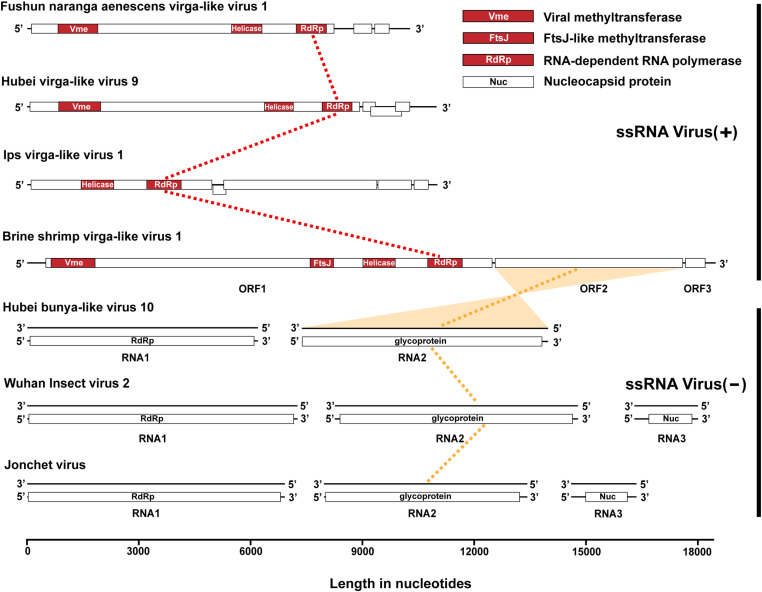
Schematic genome structure of BSVV1 and related viruses. Vmethyltransf: viral methyltransferase, FtsJ: ftsJ-like methyltransferase, RdRp: RNA-dependent RNA polymerase, Nuc: nucleocapsid protein.

The complete genome sequence of BSVV1 comprised 5’ and 3’ untranslated regions (UTRs) and three open reading frames (ORFs) with lengths of 11973 nt (ORF1, 497-12469), 5028 nt (ORF2, 12554-17581), and 528 nt (ORF3, 17659-18186), respectively (Fig 1B). To functionally annotate BSVV1, we performed a BLASTx search against the non-redundant protein sequence database available on GenBank. As expected, ORF1 was most closely related to Ips virga-like virus 1 (accession no. WPV71128.1) with 44.09% amino acid identity in RdRp. ORF1 was predicted to be transcribed in a positive-sense orientation, encoding a viral methyltransferase (635-1816), ftsJ-like methyltransferase (7586-8215), viral RNA helicase (9002-9868), and RdRp (10742-11668) by CD-search (Fig 2). These conserved functional domains were also found in known members of the *Virgaviridae* and virga-like viruses, again confirming BSVV1 as a positive-sense, single-stranded RNA virus.

We further compared the RdRp proteins of BSVV1 with related viruses from the order *Martellivirales*. The conserved palm subdomain of RdRp, including motifs A, B, and C, was extracted from these viruses (S1 Fig). Analysis revealed several highly conserved amino acid sequences across the three motifs between BSVV1 and representative viruses of the *Martellivirales*. BSVV1 shared the highest amino acid identity with Ips virga-like virus 1 in the three motifs (51.75%), followed by Hubei virga-like virus 9 (accession no. YP_009336553.1, 46.51%) and Fushun naranga aenescens virga-like virus 1 (accession no. UHM27606.1, 46.07%).

In marked contrast, the top BLASTx hit for ORF2 of BSVV1 was a putative glycoprotein of Hubei bunya-like virus 10 – a negative-sense RNA virus – with 31.92% amino acid identity. Of note, this similarity was estimated between the protein encoded by BSVV1 ORF2 transcribed in a positive-sense orientation and the protein encoded by the reverse complement sequence of the M segment of Hubei bunya-like virus 10 (Fig 2). No BLASTx hit for ORF3 was found.

**Fig 3:**
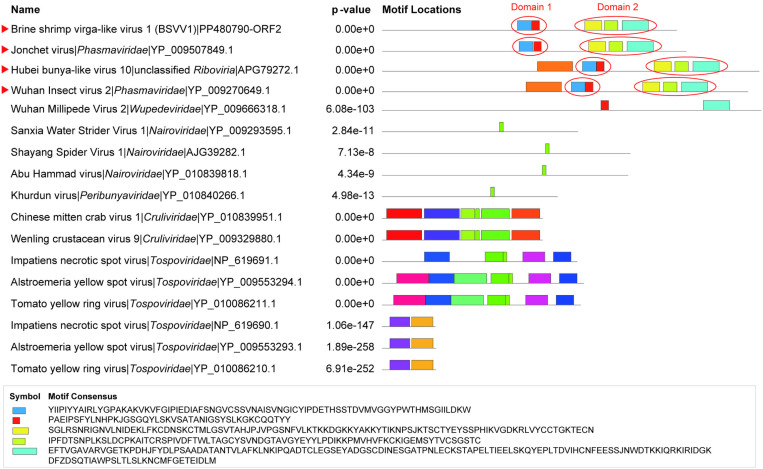
Predicted motifs of ORF2 of BSVV1 and representative members of the order *Bunyavirales.* The representative members of the *Bunyavirales* used in this analysis included the viruses most closely related to BSVV1 and those from different families within the *Bunyavirales* (downloaded from GenBank). The motifs were predicted using the online service provided by MEME. Different colors represent different functional motifs.

Although no known conserved domains were predicted in either ORF2 or ORF3 by CD-search, through MEME analysis [22] we identified five conserved motifs in the protein encoded by BSVV1 ORF2 (Figs 3, S2, S3, and S4). Interestingly, these five motifs were also found in the protein encoded by the M segment of Hubei bunya like virus 10 (accession no. KX884783.1), as well as Wuhan Insect virus 2 (accession no. NC_030750.1), and Jonchet virus (accession no. NC_038708.1), both of which are members of the order *Bunyavirales* (family *Phasmaviridae*) (Fig 3). According to the relative positions of the five motifs, they were tentatively defined as two domains for simplicity, with domain 1 including motifs 1-2 and domain 2 comprising motifs 3-5 (Fig 3). In both domains, BSVV1 shared the highest amino acid identities with Hubei bunya like virus 10 (47.20% and 37.37%), followed by Wuhan Insect virus 2 (47.02% and 32.63%) and Jonchet virus (38.67% and 34.60%). We therefore concluded that the protein encoded by BSVV1 ORF2 was related to the glycoprotein found in several viruses from the *Bunyavirales*.

A total of 26 complete or near-complete genome sequences of BSVV1 were obtained, including 25 genome sequences of BSVV1 and one (BSVV1-IT/36) that did not contain ORF3. The nucleotide sequence identities across these 25 genomes ranged from 94.75% to 99.82%. For the 26 ORF1 and ORF2 sequences, the nucleotide identities ranged between 95.01%-99.97% and 96.36%-99.98%, respectively. The amino acid sequence identities for the two ORFs were between 96.67%-99.95% for ORF1 and 98.81%-99.94% for ORF2. In addition, we compared domain 1 and domain 2 of ORF2 among the 26 BSVV1 consensus sequences, with the BSVV1 strain KAZ-SL-20 (KA/20) serving as the reference. Both domain 1 and domain 2 of ORF2 were conserved among all BSVV1 isolates, with few amino acid substitutions (S5 and S6 Figs).

### Phylogenetic analysis of BSVV1

**Fig 4:**
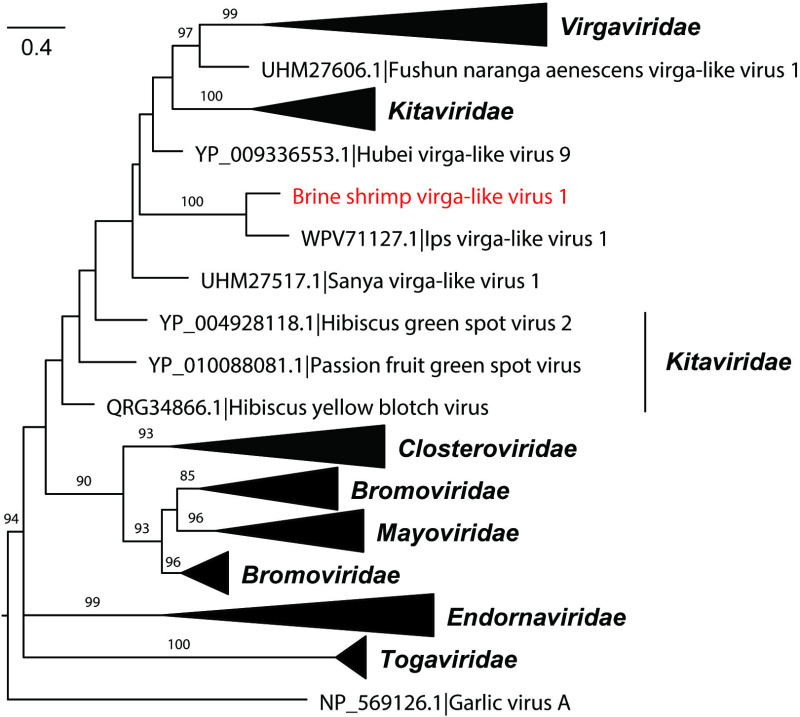
Phylogenetic analysis of the conserved palm subdomain of BSVV1 and representative members of the order *Martellivirales.* The phylogeny was estimated using the palm subdomain of RdRp protein sequences of BSVV1 and representative viruses from the *Martellivirales*. The reference sequences were downloaded from GenBank and aligned using Mafft. Phylogenetic analysis was performed using IQ-TREE, with the best-fit LG+F+I+G_4_ amino acid substitution model and 1000 bootstrap replicates. The scale bar represents the number of amino acid substitutions per site, and only bootstrap values >80% are shown.

To determine the evolutionary relationships of BSVV1, we performed phylogenetic analyses of the conserved palm subdomain of the RdRp protein (covering motifs A, B, and C) of BSVV1 and representative *Martellivirales*, previously classified by the International Committee on Taxonomy of Viruses (ICTV). Although Ips virga-like virus 1 has been classified within the *Virgaviridae*, our phylogenetic analyses revealed that it did not fall within the *Virgaviridae* (Fig 4). Rather, BSVV1 and Ips virga-like virus 1 clustered together, forming a separate branch that did not fall within any of the ICTV-approved families of the *Martellivirales*. In addition, it was notable that the family *Kitaviridae* was not monophyletic in our phylogeny, which also merits further investigation (Fig 4).

### Determination of the viral proteins of BSVV1

**Fig 5:**
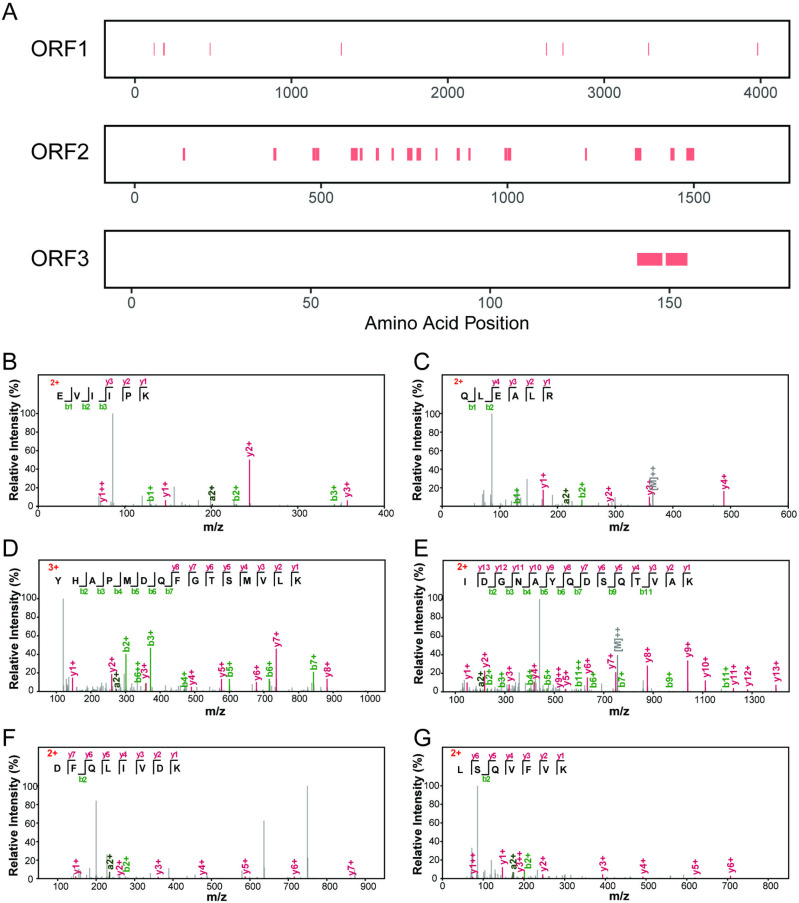
Integrating analysis of mass spectrometry data of viral ORF1, ORF2, and ORF3 protein products of samples 1, 2, and 3 of the BSVV1 positive pool LC20181207. (A) Primary sequence coverage map of ORF1, ORF2, and ORF3 protein products, with the identified peptides highlighted in red. (B, C) Annotated MS/MS spectrometry of representative peptides from the ORF1 protein products. (D, E) Annotated MS/MS spectrometry of representative peptides from the ORF2 protein products. (F, G) Annotated MS/MS spectrometry of peptides from the ORF3 protein products.

To confirm the presence of the viral proteins of BSVV1, we used mass spectrometry (MS) to identify virus-specific proteins in the sample LC20181207. With a false-positive rate of <1% in both protein and peptide identification, we successfully identified 8, 21, and 2 non-redundant tryptic peptides derived from the ORF1, 2, and 3-encoded proteins of BSVV1 (all transcribed in a positive-sense orientation), respectively, and covering 1.3%, 12.2%, and 8.6% of the full-length deduced protein sequences from different extracts of LC20181207 (named as samples 1-3 in the Materials and Methods) (Fig 5A). For the protein products of ORF1 and ORF2, the distribution of the peptide positions was uniform, covering various viral protein domains (Fig 5A). Furthermore, the representative MS/MS spectrometry yielded a continuous series of fragment ions, indicating good quality and reliable peptide identification (Fig 5B and 5G). Based on the assumption that a protein can be confidently identified by at least two peptides (i.e., the “two peptide rule” [23]), our results revealed the presence of the protein products encoded by the three ORFs of BSVV1.

### Establishment of real-time RT-PCR assay and amplification of BSVV1 in brine shrimp

We developed a BSVV1-specific TaqMan probe-based real-time RT-PCR assay, which demonstrated both high specificity and sensitivity. The specificity testing indicated that the specific amplification only occurred in the RNA extracted from BSVV1 positive brine shrimp samples, with no amplification observed in BSVV1 negative brine shrimp samples, nor in samples carrying Macrobrachium rosenbergii nodavirus (MrNV), yellow head virus genotype 8 (YHV-8), and covert mortality nodavirus (CMNV). Sensitivity testing revealed that the detection limit of the assay was at 10^2^ copies/µL. To investigate the replication of BSVV1, we hatched the BSVV1 positive brine shrimp cysts of LC20181207 and measured the viral load using the newly developed RT-qPCR assay. The viral load in the decapsulated brine shrimp was measured at 2.42×10^3^ copies/ng on the third day post-inoculation. As the nauplii transitioned through molting into post-nauplii and eventually matured into adults, the viral load within brine shrimp progressively increased. By day 14 of brine shrimp development, the viral load reached 2.07×10^6^ copies/ng, demonstrating the proliferation of BSVV1 within the brine shrimp population (S7 Fig).

### Transmission electron microscopy of brine shrimp cyst carrying BSVV1

We employed transmission electron microscopy (TEM) to examine brine shrimp cyst carrying BSVV1. Our analysis revealed the presence of virus-like particles within cytoplasmic inclusions on ultrathin sections of the cysts (S8A-D Fig). The particles were observed to be spherical in shape with an approximate diameter of 50 nm. Notably, no comparable virus-like particles were detected in the ultrathin sections of brine shrimp cysts negative for BSVV1 (S8E-F Fig).

## Discussion

Positive-sense and negative-sense RNA viruses employ different transcription and replication strategies to generate progeny virions. As such, positive-sense and negative-sense RNA viruses have traditionally been considered to be genetically and phylogenetically distinct and to have diverged in the distant evolutionary past. As a consequence, they have been classified into different viral phyla. However, in the present study we report a novel positive-sense virga-like virus – BSVV1 – in brine shrimp that contained an ORF2 transcribed in the positive-sense yet related to the glycoprotein of several negative-sense bunyaviruses, and hence indicative of an ancient recombination event between positive-sense and negative-sense RNA viruses.

The BSVV1 genome comprised three ORFs. ORF1 shared homology with the RdRp of Ips virga-like virus 1, while ORF2 encoded a protein that was related to a putative glycoprotein found in the negative-sense virus Hubei bunya-like virus 10, sharing five conserved motifs. We therefore propose that the ORF2 might have been derived from a bunyavirus via a recombination event when the bunyavirus was replicating its positive-sense strand, or via a non-replicative recombination event [24,25]. In addition, as this recombinant genome structure was found in all BSVV1 samples sequenced, representing multiple continents, and because the levels of amino acid sequence similarity in the ORFs were low, it is clear that the recombinant event itself likely occurred in the deep evolutionary past, although the exact timescale is uncertain [26]. In addition, no identifiable homologs or conserved structural motifs were identified in ORF3 of BSVV1. Further research is needed to elucidate the functions of these ORFs in the virus’s life cycle.

We used TEM to examine the morphology of viral particles. We observed a type of non-enveloped, putative viral particle measuring approximately 50 nm in size within the brine shrimp cysts containing BSVV1. However, the absence of an envelope in intracellular viral particles does not necessarily mean that the virus lacks an envelope. Bunyavirus glycoproteins play a crucial role in the formation of the viral envelope and the budding process of viral particles, interacting with the host cell membrane to facilitate the formation of the viral envelope and the release of viral particles [27,28]. However, even in the presence of a glycoprotein precursor, some bunyaviruses may not necessarily form an envelope [29,30]. Further experiments are therefore necessary to determine the presence or absence of the envelope in BSVV1.

Interestingly, our results also revealed a global distribution of BSVV1 within brine shrimp populations. The reasons behind the observed distribution patterns of BSVV1, and to what extent this was human mediated, remain a mystery in its ecological narrative and require further investigation.

In summary, the distinct genome sequence of BSVV1 from brine shrimp provided strong evidence for a recombination event involving different RNA virus phyla characterized by very different forms of genome organization, in turn providing novel insights into the emergence, diversity, and evolutionary mechanisms of RNA viruses. Future investigations should prioritize understanding the functional implications of the protein products within BSVV1.

## Materials and methods

### Ethics statement

The animal experiments conducted here were approved by the Institutional Animal Care and Use Committee of Yellow Sea Fisheries Research Institute, Chinese Academy of Fishery Sciences (approval number YSFRI-2019007). The collection and processing of brine shrimp samples were performed in strict accordance with the Guidelines of the Institutional Animal Care and Use Committee.

### Sample preparation

A total of 144 batches of brine shrimp cysts were collected from six continents, encompassing 21 countries and over 100 geographic locations worldwide. Sample LC20181207 was acquired through commercial purchase. With the exception of LC20181207, information on all the other 143 samples has been described previously [21]. Sample preparation and RNA extraction were performed as described by Dong et al. [21,31].

### Genome sequencing and validation

Library preparation of total RNA was performed according to the standard protocol provided by Illumina as previously described [21,31]. Meta-transcriptomic sequencing was performed by Novogene (Beijing, China). The raw sequencing reads were quality-controlled and *de novo* assembly was performed using Trinity [32]. Subsequently, the assembled contigs were translated and compared against the non-redundant database by BLAST to identify potential viral contigs [33]. The reads were then mapped to the viral contigs with Bowtie 2 to obtain the consensus sequence [34]. The genome sequence of BSVV1 was confirmed by Sanger sequencing using primers (S2 Table) designed based on the consensus sequence. To verify the integrity of the genome, we designed primers F23\R23 spanning ORF1, ORF2, and ORF3, and added NotI and XhoI restriction sites to the 5’ of the forward and reverse primers, respectively. PCR amplification was performed using the Phusion High-Fidelity DNA Polymerase kit (Thermo Scientific), and the product was purified using the E.Z.N.A. Gel Extraction Kit (OMEGA). The purified product and the pET-28a vector were digested with NotI and XhoI (New England Biolabs). After purification of the digested product, the product was ligated with the vector using DNA Ligation Kit (TaKaRa) and transformed into Stbl3 competent cells. After culture at 30°C for 16 h, positive clones were selected and sequenced. The 5’ and 3’ ends of the BSVV1 genome were determined using the SMARTer RACE 5’/3’ kit (Takara) according to the manufacturer’s manual. The prevalence of BSVV1 was investigated based on the results of the meta-transcriptomic sequencing. The BSVV1 consensus sequence obtained from the library KAZ-SL-20 was used as a reference genome to map the sequencing reads of all the libraries using Bowtie 2, and only genome sequences with ≤2% degenerate bases were kept [35].

### Establishment of BSVV1 TaqMan probe-based real-time RT-PCR assay

Primer-BLAST [36] was used to design specific primers and a TaqMan probe based on the ORF1 of the BSVV1 genome sequence. The specific primers BSVV1-F (5’-TCTTTATTAGACTTAACCAGCGGCT-3’) and BSVV1-R (5’-TTTCGTCTATGTAGTCCGAGACAAA -3’) were used to amplify a 73 bp amplicon. The TaqMan probe (BSVV1-P: 5’-ACAACGACCCTGCTGTTGACGTT -3’) was labelled with 6-carboxyfluorescein (FAM) at the 5’ end and N, N, N, N-tetramethyl-6-carboxyrhodamine (TAMRA) at the 3’ end. The one-step TaqMan-RT-qPCR reactions were performed in a 20 μL reaction mixture comprising 12.5 μL Probe 1-step RT-qPCR 5G Premix (TOROIVD, China), 0.5 μM of each primer (BSVV1-F/R), 0.25 μM BSVV1-P, 1 μL template RNA, and 4 μL nuclease-free water. Amplification was performed as follows: 5 min reverse transcription at 52°C and initial denaturation for 1 min at 95°C, 40 cycles of 3 s at 95°C, and 30 s at 60°C. Amplification, detection, and data analysis were performed using a CFX-96 Quantitative Fluorescence Instrument (Bio-Rad, USA).

To assess the specificity of the assay, we used total RNA extracted from brine shrimp samples that were both positive and negative for BSVV1, as well as from samples carrying MrNV, YHV-8, and CMNV as templates in a real-time RT-PCR assay. To assess the sensitivity of the assay, we cloned the cDNA of the target fragment of the ORF1 in BSVV1 and the T7 transposon sequence into a pUC57 vector. The plasmid inserted with the target fragment and the T7 transposon sequence was extracted and used as the template for *in vitro* transcription using the *In vitro* Transcription T7 Kit (TaKaRa, China) following the manufacturer’s instructions. To determine the analytical sensitivity, we used the 10-fold dilution of BSVV1 standard RNA (1.00 × 10^1^ –1.00 × 10^10^ copies/μL) as templates for real-time RT-PCR.

### Genome annotation and phylogenetic analyses

Annotation of the viral genome was performed using CD-Search [37,38]. Virus reference sequences related to those obtained here were downloaded from the NCBI non-redundant protein database. The palm subdomain (covering motifs A, B, and C) of the RdRp amino acid sequences from BSVV1 and reference viruses was identified using Palmscan [39]. The potential conserved motifs in ORF2 and ORF3 of BSVV1 were predicted using MEME v5.5.5 (https://meme-suite.org/meme/tools/meme). The parameters of MEME were set to discover 20 motifs with a minimum width of 6 resides and maximum width of 200 residues. For all the motifs, WebLogo v3.7.12 was used to estimate and visualize the amino acid sequence logos [40]. Pairwise amino acid identities between BSVV1 and reference viruses were estimated using BLASTp v2.9.0+ [33].

To determine the phylogenetic relationships of BSVV1, we performed multiple sequence alignment using MAFFT v7.520 [41] with ambiguously aligned regions removed using trimAl v1.4.rev15 [42]. Phylogenetic analysis and model selection were performed using IQ-TREE v2.2.5 [43], with substitution models chosen by the Bayesian information criterion (BIC), and employing 1000 bootstrap replicates. Phylogenetic trees were rooted and visualized using FigTree v1.4.4.

### Viral preparation with differential centrifugation

The brine shrimp sample (BSVV1 positive sample LC20181207) was transferred into a 50 mL centrifuge tube, with 30 ml of Histidine-Penaeid Physiological Buffer (His-PPB) (376.07 mM NaCl, 6.32 mM K_2_SO_4_, 14.41 mM CaCl_2_, 6.4 mM MgSO_4_, and 26.1 mM Histidine, pH 6.5) [44]. The brine shrimp tissues were homogenized at a speed of 10,000 rpm in an ice bath for 10 s. 200 μL of the homogenate (named sample 1) was used for LC-MS/MS analysis. The remainder of the homogenate was then centrifuged at 7000 rpm for 15 m at 4°C, and the supernatant was stored at 4°C. 30 mL of His-PPB was added to the pellet, which was homogenized at 10000 rpm in an ice bath for 10 s, and was subsequently centrifuged at 5,000 rpm for 10 m at 4°C, further separating the supernatant and the pellet. The supernatant was stored at 4°C. The above steps were repeated twice, with all the supernatants combined and filtered through a 0.45 μm filter membrane. 200 μL of the filtered solution (named sample 2) was also used for LC-MS/MS analysis. The filtered solution was centrifuged at 100000 g for 2 h at 4°C to precipitate viral particles. Finally, 400 μL of His-PPB was added to re-suspend the pellet, and 200 μL of the re-suspended solution (named sample 3) was simultaneously used for LC-MS/MS analysis.

### Validation of the viral proteins of BSVV1

For the three samples prepared as described above, the Solvent Precipitation SP3 (SP4) procedure was used for protein digestion [45]. Briefly, ~50 μg of lysates (1 μg/μL in 2% SDS, 100 mM Tris-HCl, and 100mM Dithiothreitol, pH 7.6) were alkylated with 200 mM iodoacetamide for 30 m in the darkness. Silica beads/glass spheres (9 – 13 μm mean particle diameter; Sigma catalogue no. 440345) were suspended at an initial concentration of 100 mg/mL in Milli-Q water, washed sequentially with 100% acetonitrile, 100 mM ammonium bicarbonate (ABC), twice with water, and pelleted at 16000g for 1 min, and the supernatant was discarded (also removing any unpelleted beads). Glass beads were adjusted to a final concentration of 50 mg/mL in water. According to a 10:1 bead/protein ratio, glass beads were added to lysates and gently mixed at 800 rpm. Next, 4 volumes of 100% acetonitrile were added, and tubes were mixed for 5 s at 800 rpm. Samples were centrifuged for 5 min at 16000 ×g at 4°C. The supernatants were aspirated and carefully washed three times with 80% ethanol by 2 min centrifugation at 16000 ×g. Protein aggregates were digested at 37°C for 18 h with trypsin (Promega) at a concentration of 1:50 (w/w) in 50 mM ABC. Peptides were desalted by C18 tip and dried using Speed-Vac (Thermo Scientific).

An LC-MS/MS analysis was performed on a Q-Exactive HF mass spectrometer (Thermo Fisher Scientific, USA) connected to an online nanoflow EASY nLC1200 HPLC system (Thermo Fisher Scientific, USA). Approximately 1 μg peptides of each sample were resolved in 0.1% formic acid and loaded on a self-packed column (75 μm × 200 mm, 3 μm ReproSil C18 beads, Dr. Maisch GmbH, Germany). The peptides were then separated with a 60 m gradient at a flow rate of 300 nL/min using solvent A (0.1% formic acid in H_2_O) and solvent B (0.1% formic acid in 80% acetonitrile). The 60 m gradient was set as follows: 1%-4% B in 1 m; 4%-26% B in 43 m; 26%-32% B in 5 m; 32%-90% B in 2 m; 90% B for 9 m. The spray voltage was set at 2.3 kV in positive ion mode and the capillary was set at 300°C. Data-dependent acquisition was performed using the Xcalibur software. The full MS resolution was set as 60,000 for full MS scan (150-1700 m/z) and 15,000 for MS/MS scan. The automatic gain control (AGC) targets of the ion trap were set to 3 e6 for full MS scan and 1 e5 for MS/MS scan. The maximum ion injection time was set to 20 ms for full MS scan, and 100 ms for MS/MS scan. The normalized collision energy (NCE) was set at NCE 27% and the dynamic exclusion time was 30 s. The top 20 data-dependent MS/MS scans were acquired by high-energy collision dissociation (HCD). The isolation window was set to 1.2 m/z.

Raw mass spectrometry data was analyzed using the pFind software version 3.2.0 against the amino acid sequences derived from ORF1, ORF2, and ORF3 of BSVV1 with a false discovery rate (FDR) < 0.01 at the levels of peptides and proteins, respectively. Other settings for peptide identification were: Carbamidomethyl (C) as a fixed modification, Oxidation (MW) and Deamidation (NQ) as variable modifications, and trypsin/P as the digestive enzyme.

### Transmission electron microscopy of brine shrimp cyst carrying BSVV1

Based on the results of the meta-transcriptomic analysis [21], we selected two brine shrimp cysts (1106 and 1538) that did not carry the BSVV1 as well as two others (CHN-AQ-HN-1 and KAZ-SL-20) carrying a high copy number of BSVV1 virus, while minimizing the presence of other viruses for TEM observation. The deshelled brine shrimp cyst samples were fixed in a TEM holder at 4°C. Ultrathin sections were prepared on collodion-coated grids using the previously published procedure [46]. All grids were examined using a JEOL JEM-1400 Flash electron microscope (Jeol Solutions for innovation, Peabody, MA, USA) at an operating voltage of 80 KV.

### Hatching and breeding of brine shrimp

A total of 0.3 g of brine shrimp cysts (LC20181207) was added to an incubation tube containing 600 mL of natural seawater, and was incubated under conditions of 28°C, salinity of 30 g/L, pH=8, light intensity of 2000 lx, and continuous aeration. After incubation for about 24 h, the nauplii were collected and placed in 1 L of seawater, and were cultivated under conditions of 28°C, salinity of 30 g/L, pH=8, light intensity of 2000 lx, and continuous aeration. Brine shrimp were fed twice a day with a suspension of 2 g of Chlorella powder in 50 mL of seawater, with 50% water change every 3 days. When the brine shrimp grew to a body length of ~1 cm, it was collected and stored at -80°C for further use.

## Supporting information

S1 Table Host, geographic, and meta-transcriptomic information for brine shrimp virga-like virus 1 (BSVV1) positive pools.
Definition of the parthenogenetic *Artemia* lineage was after Asem et al., 2024 [47].
(PDF)

S2 Table Primers designed to validate the presence of brine shrimp virga-like virus 1 (BSVV1). (PDF)

S1 Fig Sequence logo of the predicted motifs A, B, and C of RdRp of BSVV1. Generated using WebLogo.
(PDF)

S2 Fig Sequence logo of domain 1 encoded by ORF2 of BSVV1 and the M segment of the three related bunyaviruses. Generated using WebLogo.
(PDF)

S3 Fig Sequence logo of domain 2 encoded by ORF2 of BSVV1 and the M segment of the three related bunyaviruses. Generated using WebLogo.
(PDF)

S4 Fig Sequence alignment of domain 1 and 2 encoded by ORF2 of BSVV1 and the M segment of the three related bunyaviruses. Generated using Mafft.
(PDF)

S5 Fig Multiple sequence alignment of domain 1 of the BSVV1 sequences. The sequences of BSVV1 from different geographic regions were aligned using MAFFT. The BSVV1 isolate KA/20 was utilized as the reference sequence. Identical amino acids are indicated by “.”, while different amino acids are displayed with different colored backgrounds. Sequence names are shown on the left, with the amino acid positions indicated below each alignment block.
(PDF)

S6 Fig Multiple sequence alignment of domain 2 of the BSVV1 sequences. The sequences of BSVV1 from different geographic regions were aligned using MAFFT. BSVV1 isolate KA/20 was utilized as the reference sequence. Identical amino acids are indicated by “.”, while different amino acids are displayed with different colored backgrounds. Sequence names are shown on the left, with the amino acid positions indicated below each alignment block.
(PDF)

S7 Fig Dynamic change of BSVV1 viral load with the development of brine shrimp. (PDF)

S8 Fig Transmission electron micrographs of the putative BSVV1 particles. (A, B, C, and D) Brine shrimp cysts carrying BSVV1. (B) The magnified view of the red frame of panel A. (D) The magnified view of the red frame of panel C. (E and F) Brine shrimp cysts negative for BSVV1.
(PDF)
