# Supplementary material for: A positive-sense single-stranded RNA virus acquired a negative-sense open reading frame through recombination
Source: PLoS Pathog. 2025 Apr 8;21(4):e1013015. doi: 10.1371/journal.ppat.1013015 (PMC11978036; doi:10.1371/journal.ppat.1013015)
Supplement: S1 Table — Definition of the parthenogenetic Artemia lineage was after Asem et al., 2024 [47]. (PDF) [file ppat.1013015.s001.pdf]

Table S1 Host, geographic, and metatranscriptomic information for brine shrimp virga-like virus 1 (BSVV1) positive pools.

NO.	GenBank accession	Sample name	Mixed	Place	Country	Abbreviation	Continent	Year	Host species	Library	Virus name/isolate	Length	Raw Reads	Viral Reads	RPKM
1	PP480751	1495	N	ND	Australia	AUS	Oceania	ND	parthenogenetic *Artemia* lineage	1495	BSVV1 isolate AU/95	18,505	76,699,482	14,876	10.48
2	PP480752	1699X	N	ND	Algeria	ALG	Africa	2007	*A. sinica*	1699X	BSVV1 isolate AL/99	18,187	79,311,790	45,900	31.82
3	PP480754	1815	N	Ebeyty	Russia	RUS	Asia	before 201	parthenogenetic *Artemia* lineage	1815	BSVV1 isolate RU/15	18,414	83,670,010	86,657	56.25
4	PP480756	1817	N	Maloye (M.) Medvezhye	Russia	RUS	Asia	before 201	parthenogenetic *Artemia* lineage	1817	BSVV1 isolate RU/17	18,416	96,992,124	27,483	15.39
5	PP480757	1818	N	Bolshoye (B.) Medvezhye	Russia	RUS	Asia	before 201	parthenogenetic *Artemia* lineage	1818	BSVV1 isolate RU/1	18,502	78,709,062	26,745	18.37
6	PP480760	CHN-SL-17	Y	Wulateqianqi, Inner Mongoli	China	CHN	Asia	1998	*A. sinica,* parthenogenetic *Artemia* lineage	CHN-NM-1	BSVV1 isolate NM/1	18,521	72,927,982	11,522	8.53
		CHN-SL-44	Y	Wulateqianqi, Inner Mongoli	China	CHN	Asia	1993							
		CHN-SL-47	Y	Wulateqianqi, Inner Mongoli	China	CHN	Asia	1998							
		CHN-SL-49	Y	Bameng, Inner Mongolia	China	CHN	Asia	2000							
		CHN-SL-55	Y	Bayan Lake, Inner Mongolia	China	CHN	Asia	2014							
7	PP480761	CHN-SL-4	Y	Naritumusu, Inner Mongolia	China	CHN	Asia	2013	*A. sinica*	CHN-NM-2	BSVV1 isolate NM/2	18,345	88,960,698	52,662	32.27
		CHN-SL-15	Y	Dahan Lake, Inner Mongolia	China	CHN	Asia	1994							
		CHN-SL-19	Y	Haolebaoqing, Inner Mongoli	China	CHN	Asia	1997							
		CHN-SL-26	Y	Haolebaoqing, Inner Mongoli	China	CHN	Asia	1998							
		CHN-SL-60	Y	Wushenqi, Inner Mongolia	China	CHN	Asia	2018							
8	PP480762	XXN-SL-7X	N	Aibi Lake, Xinjiang	China	CHN	Asia	2011	parthenogenetic *Artemia* lineage	XXN-SL-7X	BSVV1 isolate XJ/7	18,231	87,047,354	19,396	12.22
9	PP480763	CHN SL 24	N	Yuncheng Lake, Shanxi	China	CHN	Asia	1991	*A. sinica*	CHN-SL-24	BSVV1 isolate SX/24	18,405	77,945,304	18,469	12.87
10	PP480765	CHN-SL-2	Y	Mengba Lake, Xinjiang	China	CHN	Asia	2011	*A. sinica,* parthenogenetic *Artemia* lineage	CHN-XJ-2	BSVV1 isolate XJ/2	18,448	73,009,554	16,908	12.55
		CHN-SL-22	Y	Jingyu Lake, Xinjiang	China	CHN	Asia	2000							
		CHN-SL-37	Y	Jingyu Lake, Xinjiang	China	CHN	Asia	2002							
		CHN-SL-45	Y	Jingyu Lake, Xinjiang	China	CHN	Asia	2001							
		KAZ-SL-10	Y	Kezilkak Lake	Kazakhstan	KAZ	Asia	2016							
11	PP480766	CHN-SL-18	Y	Yanabeng Co, Tibet	China	CHN	Asia	2002	*A. tibetiana, A. sinica*	CHN-XZ-1	BSVV1 isolate XZ/1	18,636	72,524,546	26,278	19.44
		CHN-SL-38	Y	Unkown Saltlake, Tibet	China	CHN	Asia	1998							
		CHN-SL-48	Y	ND	China	CHN	Asia	2002							
		CHN-SL-52	Y	ND	China	CHN	Asia	2001							
		CHN-SL-67	Y	Bongo Co, Tibet	China	CHN	Asia	2019							
12	PP480770	LBY-SW-2	N	Abu Kammash	Libya	LBY	Africa	2017	*A. salina*	LBY-SW-2	BSVV1 isolate LB/2	18,399	73,346,312	14,385	10.66
13	PP480771	RUS-SL-3	N	Bolshoye Medvezhye	Russia	RUS	Asia	2014	parthenogenetic *Artemia* lineage	RUS-SL-3	BSVV1 isolate RU14	18,506	59,801,516	13,402	12.11
14	PP480774	XXA-SL-2X	N	Teke Lake	Kazakhstan	KAZ	Asia	2016	*A. sinica*	XXA-SL-2X	BSVV1 isolate KA/16	18,536	88,189,402	34,958	21.39
15	PP480775	LBY-SW-3	N	Abu Kammash	Libya	LBY	Africa	2007	*A. salina*	LBY-SW-3	BSVV1 isolate LB/3	18,232	83,863,906	43,721	28.59
16	PP480776	CHN-SL-36	N	Qixiang Co, Tibet	China	CHN	Asia	1998	*A. tibetiana*	CHN-SL-36	BSVV1 isolate TB/36	18,196	84,795,232	2,518	1.63
17	PP480777	CHN-SL-58	N	Qixiang Co, Tibet	China	CHN	Asia	2016	*A. tibetiana*	CHN-SL-58	BSVV1 isolate TB/58	18,132	59,224,454	1,942	1.81
18	PP480779	1632	N	Sebkha d' El Adhibett	Tunisia	TUN	Africa	2003	*A. salina*	1632	BSVV1 isolate TU/1	18,228	71,796,314	283,734	216.81
19	PP480783	CHN-SL-23	Y	Haiyan Lake, Qinghai	China	CHN	Asia	2001	*A. sinica, A. tibetiana*	CHN-QH-1	BSVV1 isolate QH/1	18,288	79,731,900	98,154	67.31
		CHN-SL-27	Y	Haiyan Lake, Qinghai	China	CHN	Asia	2002							
		CHN-SL-35	Y	Xiaochaidan, Qinghai	China	CHN	Asia	1992							
20	PP480784	CHN-SL-16	Y	Gahai Lake, Qinghai	China	CHN	Asia	1991	parthenogenetic Artemia lineage	CHN-QH-2	BSVV1 isolate QH/2	18,280	72,956,022	30,200	22.64
		CHN-SL-28	Y	Gahai Lake, Qinghai	China	CHN	Asia	1992							
		CHN-SL-32	Y	Gahai Lake, Qinghai	China	CHN	Asia	1992							
		CHN-SL-34	Y	Gahai Lake, Qinghai	China	CHN	Asia	1992							
21	PP480785	CHN-SL-20	Y	Dabancheng Lake, Xinjiang	China	CHN	Asia	1991	*A. sinica,* parthenogenetic *Artemia* lineage	CHN-XJ-1	BSVV1 isolate XJ/1	17,901	83,626,358	26,278	17.55
		CHN-SL-21	Y	Balikun Lake, Xinjiang	China	CHN	Asia	1992							
		CHN-SL-65	Y	Balikun Lake, Xinjiang	China	CHN	Asia	2018							
22	PP480790	KAZ-SL-20	N	Teke Lake	Kazakhstan	KAZ	Asia	2016	*A. sinica,* parthenogenetic	KAZ-SL-20	BSVV1 isolate KA/20	18,463	76,645,498	27,294	19.29
23	PP480791	RUS-SL-11	Y	Ulzal	Russia	RUS	Asia	2015	parthenogenetic *Artemia* lineage	RUS-2	BSVV1 isolate RU/2	18,165	81,786,224	48,350	32.54
		RUS-SL-13	Y	Tyumen	Russia	RUS	Asia	2015							
24	PP480793	CHN-SW-34	N	ND	China	CHN	Asia	1994	*A. sinica*	CHN-AQ-HN-	BSVV1 isolate HN/1	18,516	86,354,946	307,322	192.20
25	PP480795	1708	N	Abu Kammash	Libya	LBY	Africa	2003	*A. salina*	1708	BSVV1 isolate AK/03	18,272	93,402,356	9,678	5.67
26	PP480797	1436	N	Margherita di Savoia	Italy	ITA	Europe	before 199	parthenogenetic *Artemia* lineage	1436	BSVV1 isolate IT/36	16,744	100,366,706	13,050	7.77
