# Supplementary material for: A positive-sense single-stranded RNA virus acquired a negative-sense open reading frame through recombination
Source: PLoS Pathog. 2025 Apr 8;21(4):e1013015. doi: 10.1371/journal.ppat.1013015 (PMC11978036; doi:10.1371/journal.ppat.1013015)
Supplement: S2 Table — (PDF) [file ppat.1013015.s002.pdf]

Table S2 Primers designed to validate the presence of brine shrimp virga-like virus 1 (BSVV1).

Primers	Primer sequence 5’-3’
BSVV1-F1	GACCTTGCAAGCAGTGGTTC
BSVV1-R1	GAATTGCGTGTAAGTGGCGT
BSVV1-F2	TCAGGGCGAAAAGAGAGCAT
BSVV1-R2	ACGTGTAGAAGGCTGGCATA
BSVV1-F3	CTCTACGAGCTTTCGGGCAT
BSVV1-R3	TTCTCTTCGGCAGTCCATCC
BSVV1-F4	CGCACCAACAGGATTGTCAC
BSVV1-R4	TTTTGAACTCTCGCTCCCCC
BSVV1-F5	GTTGCAACGGAATGTGAGAGAAA
BSVV1-R5	ACTTCGCTTTTCCACAGAGC
BSVV1-F6	AGTGTGCGAGTCACGTTCTT
BSVV1-R6	AACTCTCCCAAGCCAGCAAT
BSVV1-F7	GACGGCAATGGAAGTGAACG
BSVV1-R7	AAGCTCGCTCGGGGAAAATA
BSVV1-F8	GAACGTCGTGAGAATGGGGA
BSVV1-R8	GTCGTCCTCGTCGACTTCAA
BSVV1-F9	AACTTAAGAGCACGACGCCA
BSVV1-R9	GCTTCACGATTCGCACCATC
BSVV1-F10	CGGCGTATTTCCTGGACTTG
BSVV1-R10	AGCTAGTTTGCGGTCGGATT
BSVV1-F11	TCGTCGAAATCCCCAATGCT
BSVV1-R11	CGATCGCCCAAAAGTTCCAC
BSVV1-F12	GTCCCGTACGGATGAACCTA
BSVV1-R12	CTGTAATTGCGTCTCGCTGC
BSVV1-F13	AAAAGAGCGTGCTGGGTACT
BSVV1-R13	CTGAGCGAATTCCGCTTGC
BSVV1-F14	TCCAAACGATCGCGTACCAT
BSVV1-R14	ACTGCCGAAAGTAAAGCGGA
BSVV1-F15	TACAACGACCCTGCTGTTGA
BSVV1-R15	GACAACCCCGATGTTTACGC
BSVV1-F16	CGACCTTGCCCGAATGATTTC
BSVV1-R16	CACCAACCGTGAAGTGGAGA
BSVV1-F17	GGCGATTACCTGGACGCTTA
BSVV1-R17	GTCCGGAGTGAAGAGGGTTT
BSVV1-F18	GGAAGTCGTAGCGATGCAGA
BSVV1-R18	TGAGCACCATACTCGTACCG
BSVV1-F19	TTCTCAAAGTGACCGACGCA
BSVV1-R19	CTGGGGAAAGGTGCTAACCA
BSVV1-F20	TGCGTCGCCTTCAGTCTTTA
BSVV1-R20	TTACCCAGGCAGTTGTAGGC
BSVV1-F21	CCTCTATGGGCGTGTCAGTT
BSVV1-R21	ACCGCCTCTACGCTGAATTT
BSVV1-F22	TGACGGTGACGCTTATCAGG
BSVV1-R22	TGGTCTTAACAGCCGGAGTG
BSVV1-F23	AAAGCGGCCGCTTTAAGTCGGGCCTGACCCTCTATAA
BSVV1-R23	CCGCTCGAGCGGCGGAATCGACTTTTTCG
