# Supplementary figures and images for: A positive-sense single-stranded RNA virus acquired a negative-sense open reading frame through recombination
Source: PLoS Pathog. 2025 Apr 8;21(4):e1013015. doi: 10.1371/journal.ppat.1013015 (PMC11978036; doi:10.1371/journal.ppat.1013015)

(Motifs)

A

B

C

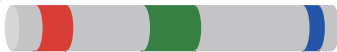

RdRp palm domain

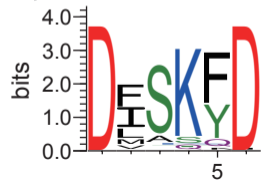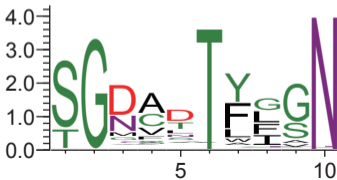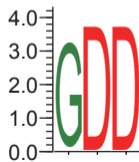

Supplement: S1 Fig — Generated using WebLogo. (PDF) [file ppat.1013015.s003.pdf]

# Domain 1

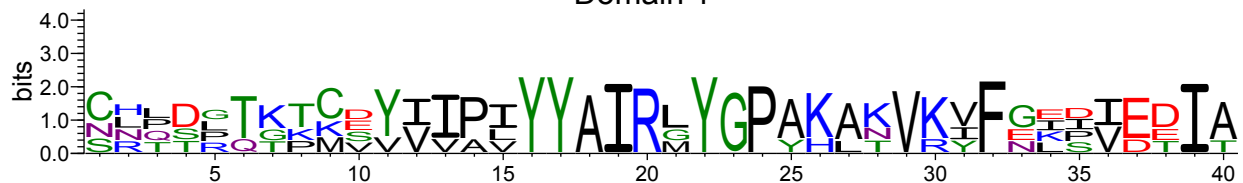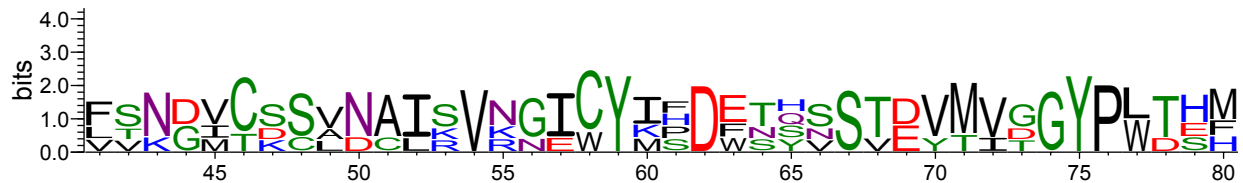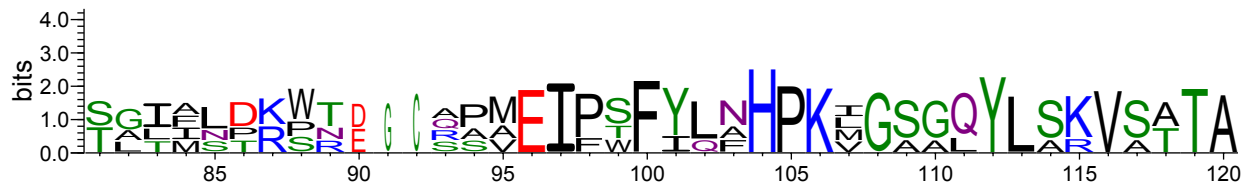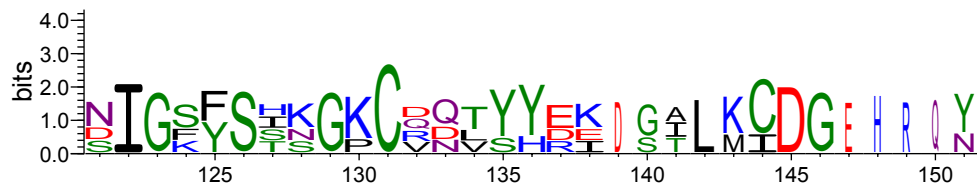

Supplement: S2 Fig — Generated using WebLogo. (PDF) [file ppat.1013015.s004.pdf]

# Domain 2

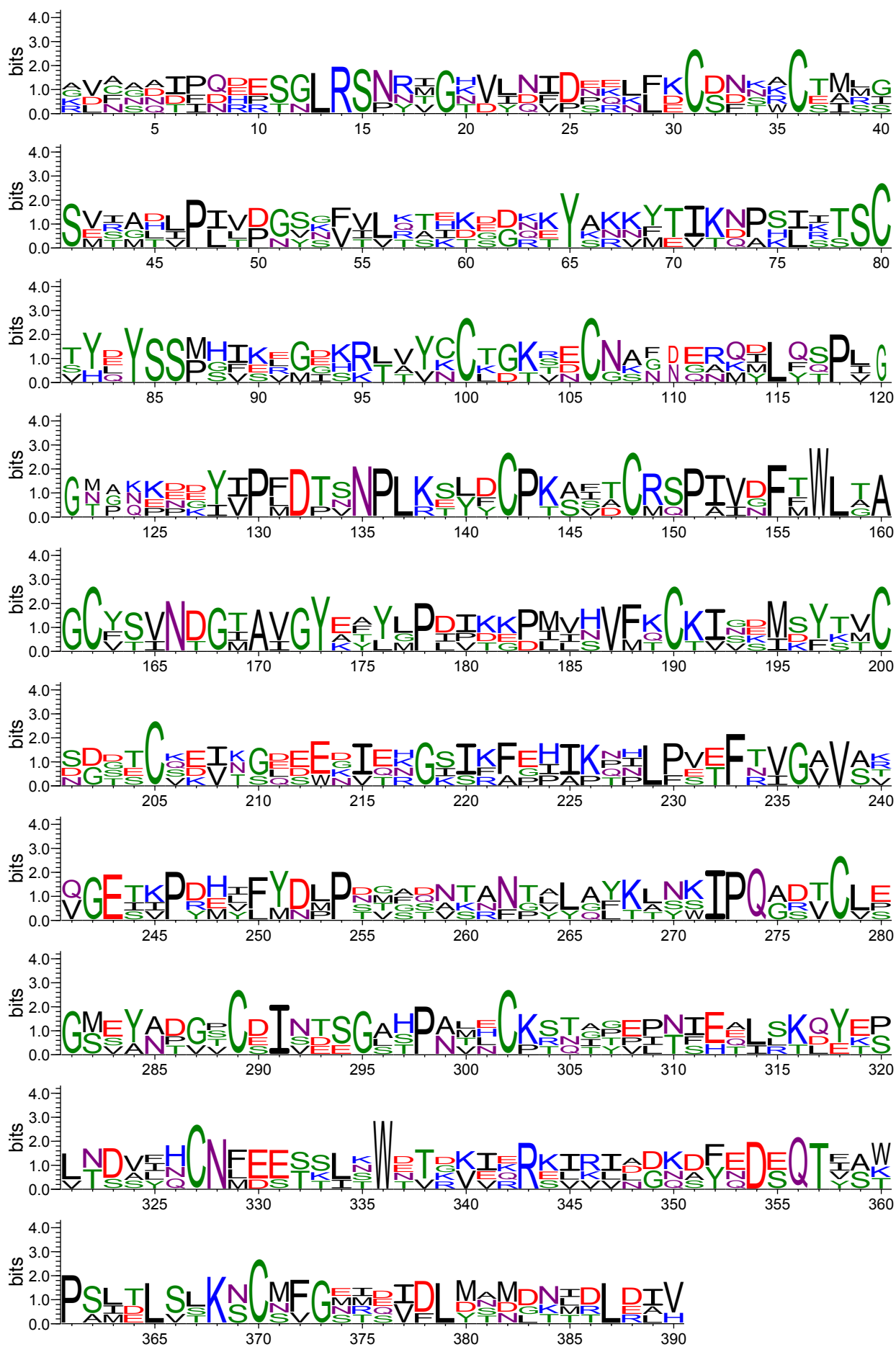

Supplement: S3 Fig — Generated using WebLogo. (PDF) [file ppat.1013015.s005.pdf]

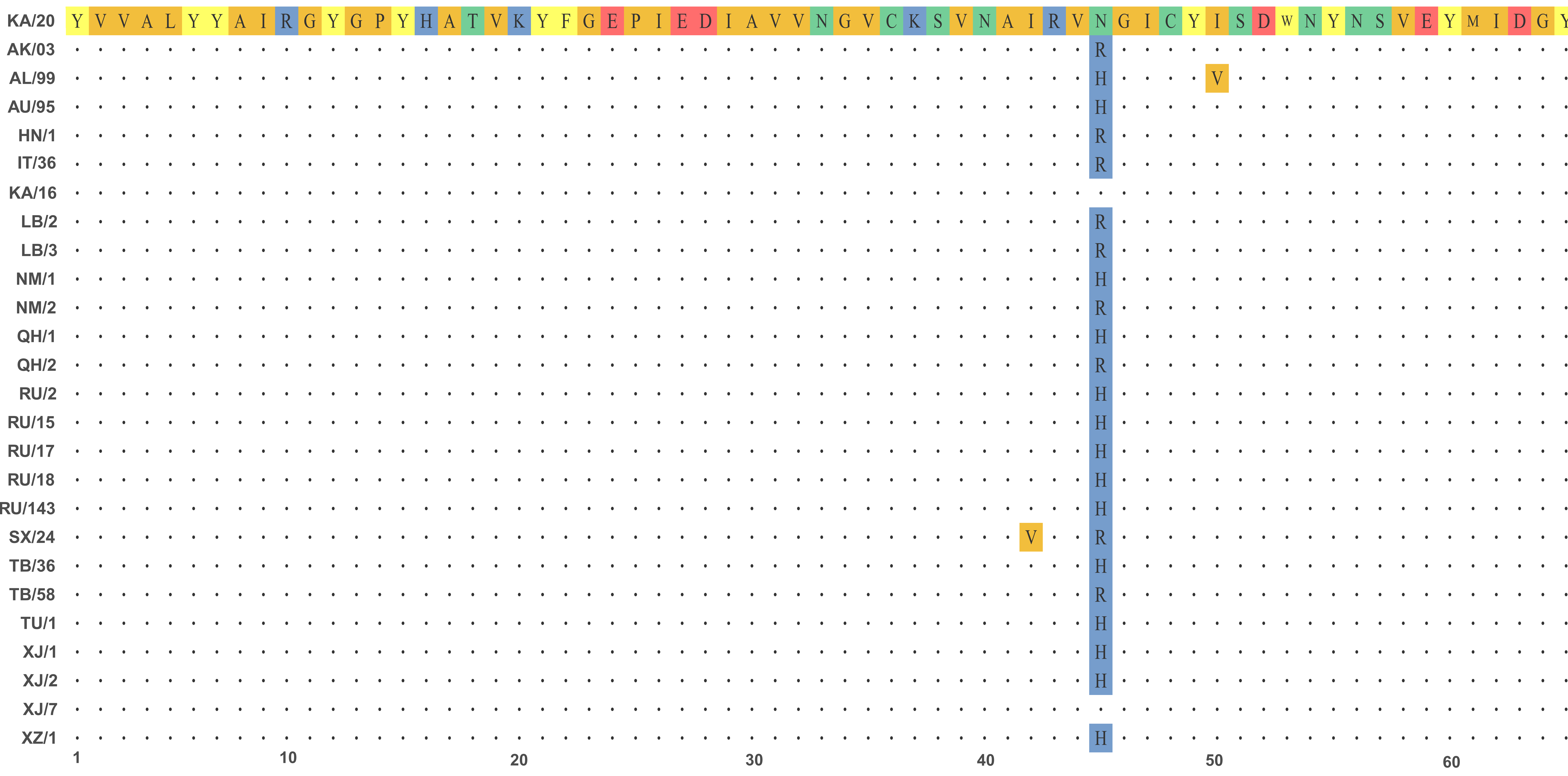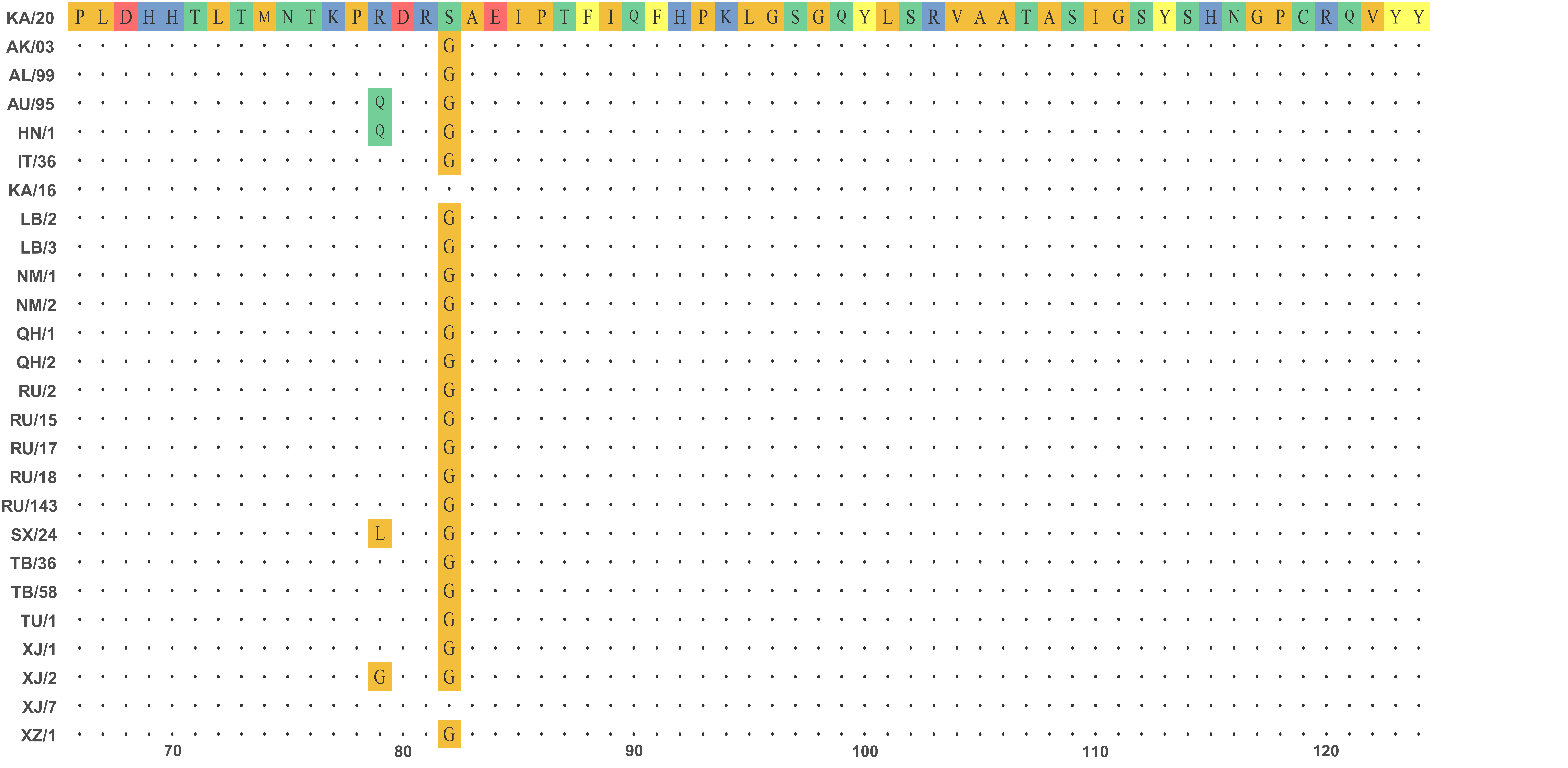

Supplement: S5 Fig — The sequences of BSVV1 from different geographic regions were aligned using MAFFT. The BSVV1 isolate KA/20 was utilized as the reference sequence. Identical amino acids are indicated by “.”, while different amino acids are displayed with different colored backgrounds. Sequence names are shown on the left, with the amino acid positions indicated below each alignment block. (PDF) [file ppat.1013015.s007.pdf]

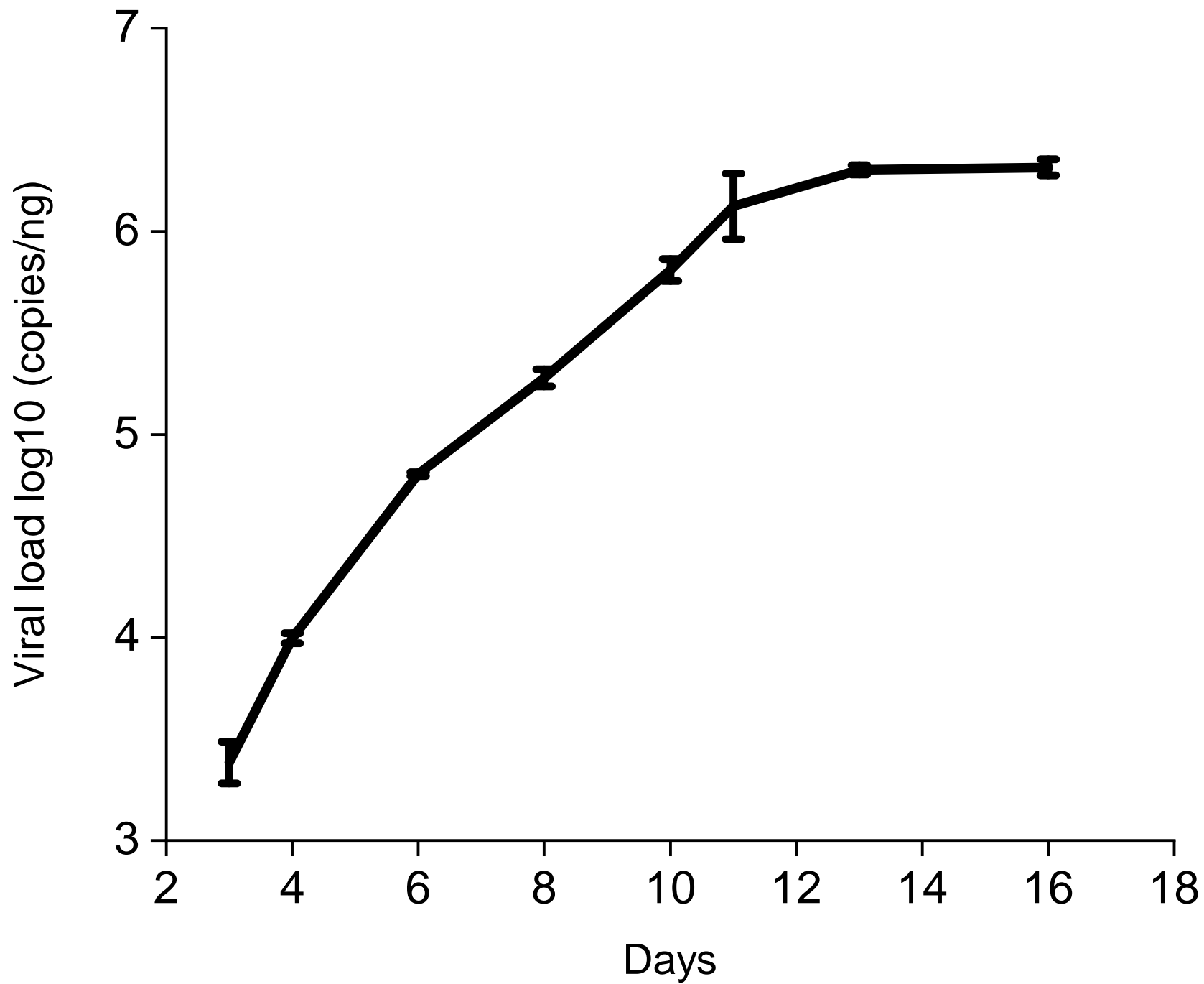

Supplement: S7 Fig — (PDF) [file ppat.1013015.s009.pdf]

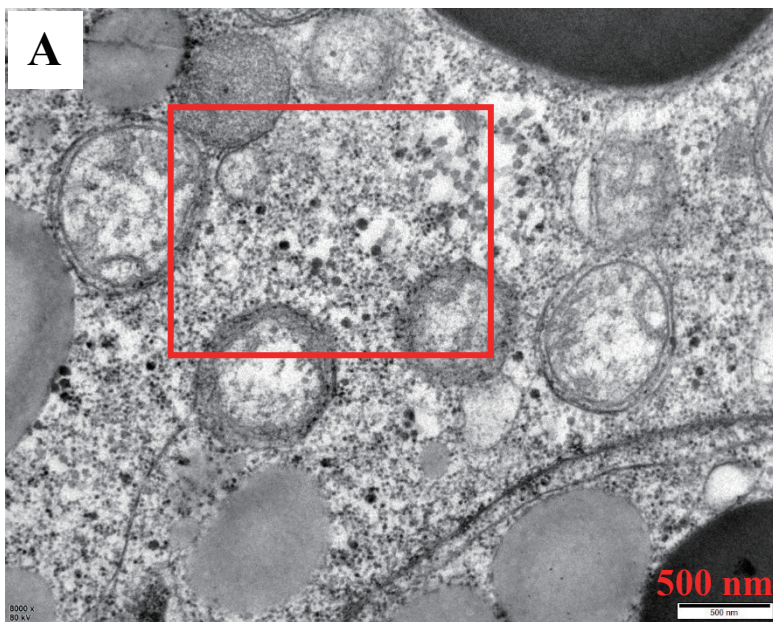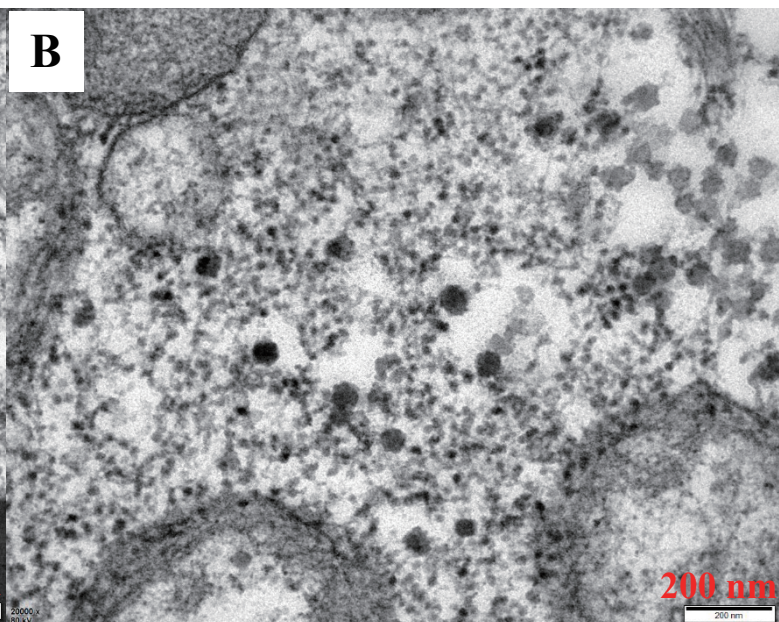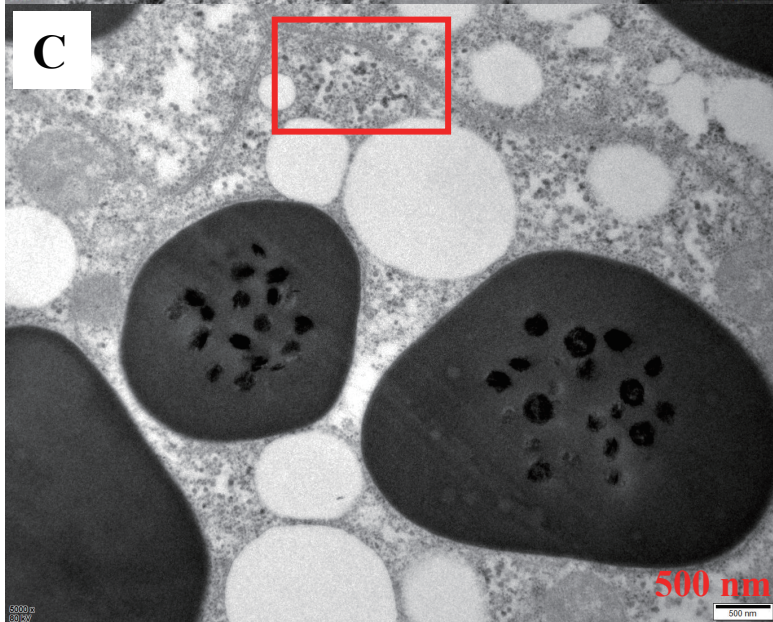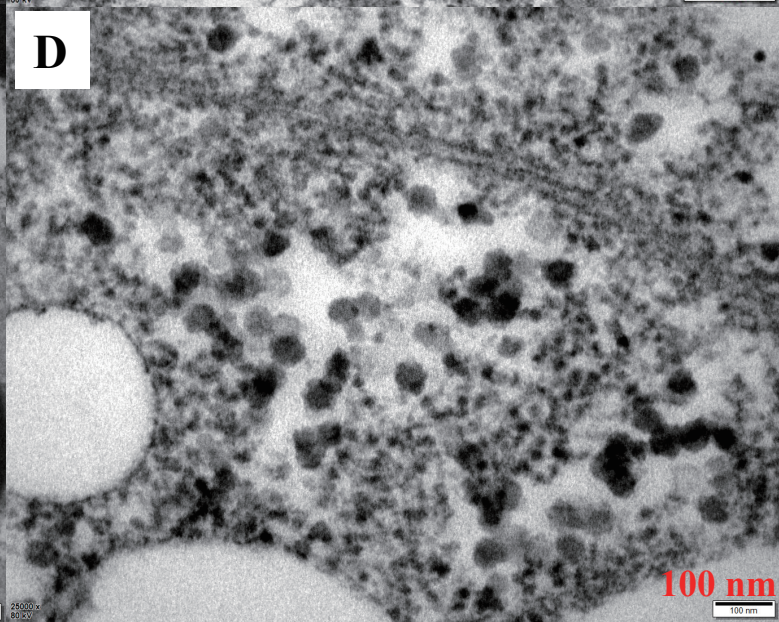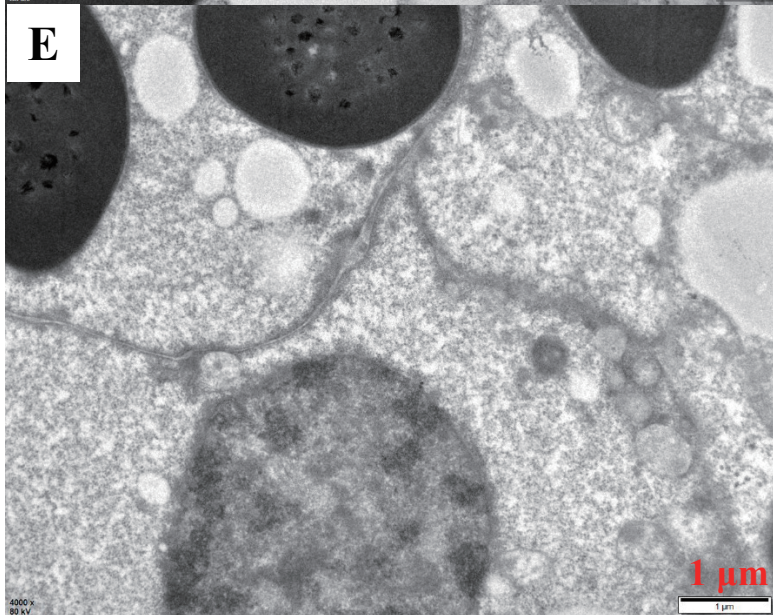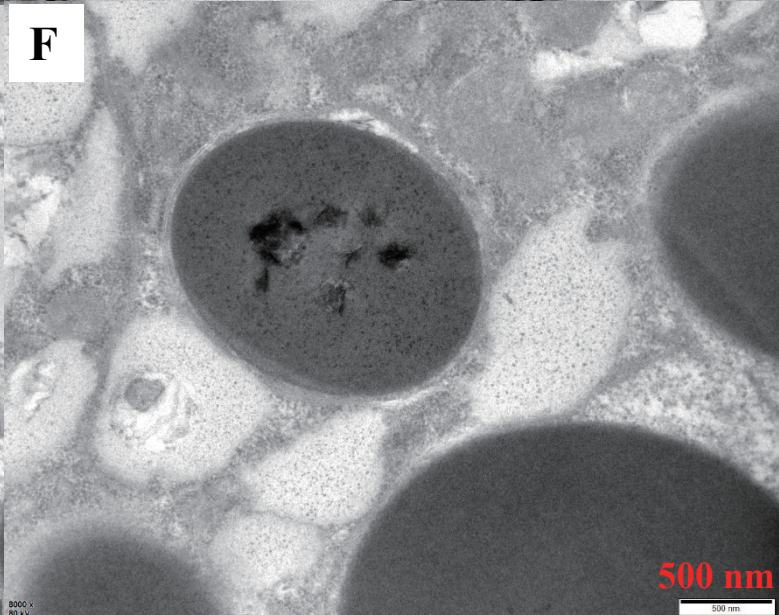

Supplement: S8 Fig — (A, B, C, and D) Brine shrimp cysts carrying BSVV1. (B) The magnified view of the red frame of panel A. (D) The magnified view of the red frame of panel C. (E and F) Brine shrimp cysts negative for BSVV1. (PDF) [file ppat.1013015.s010.pdf]
