# Supplementary material for: A positive-sense single-stranded RNA virus acquired a negative-sense open reading frame through recombination
Source: PLoS Pathog. 2025 Apr 8;21(4):e1013015. doi: 10.1371/journal.ppat.1013015 (PMC11978036; doi:10.1371/journal.ppat.1013015)
Supplement: S4 Fig — Generated using Mafft. (PDF) [file ppat.1013015.s006.pdf]

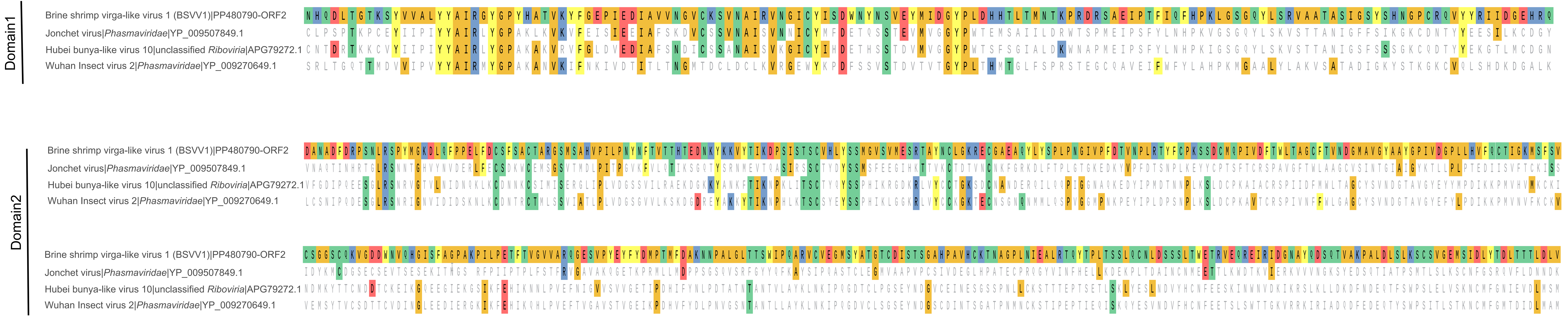

Domain1
Brine shrimp virga-like virus 1 (BSVV1)|PP480790-ORF2
Jonchet virus|Phasmaviridae|YP_009507849.1
Hubei bunya-like virus 10|unclassified Riboviria|APG79272.1
Wuhan Insect virus 2|Phasmaviridae|YP_009270649.1
Domain2
Brine shrimp virga-like virus 1 (BSVV1)|PP480790-ORF2
Jonchet virus|Phasmaviridae|YP_009507849.1
Hubei bunya-like virus 10|unclassified Riboviria|APG79272.1
Wuhan Insect virus 2|Phasmaviridae|YP_009270649.1
Brine shrimp virga-like virus 1 (BSVV1)|PP480790-ORF2
Jonchet virus|Phasmaviridae|YP_009507849.1
Hubei bunya-like virus 10|unclassified Riboviria|APG79272.1
Wuhan Insect virus 2|Phasmaviridae|YP_009270649.1
