# Supplementary material for: A positive-sense single-stranded RNA virus acquired a negative-sense open reading frame through recombination
Source: PLoS Pathog. 2025 Apr 8;21(4):e1013015. doi: 10.1371/journal.ppat.1013015 (PMC11978036; doi:10.1371/journal.ppat.1013015)
Supplement: S6 Fig — The sequences of BSVV1 from different geographic regions were aligned using MAFFT. BSVV1 isolate KA/20 was utilized as the reference sequence. Identical amino acids are indicated by “.”, while different amino acids are displayed with different colored backgrounds. Sequence names are shown on the left, with the amino acid positions indicated below each alignment block. (PDF) [file ppat.1013015.s008.pdf]

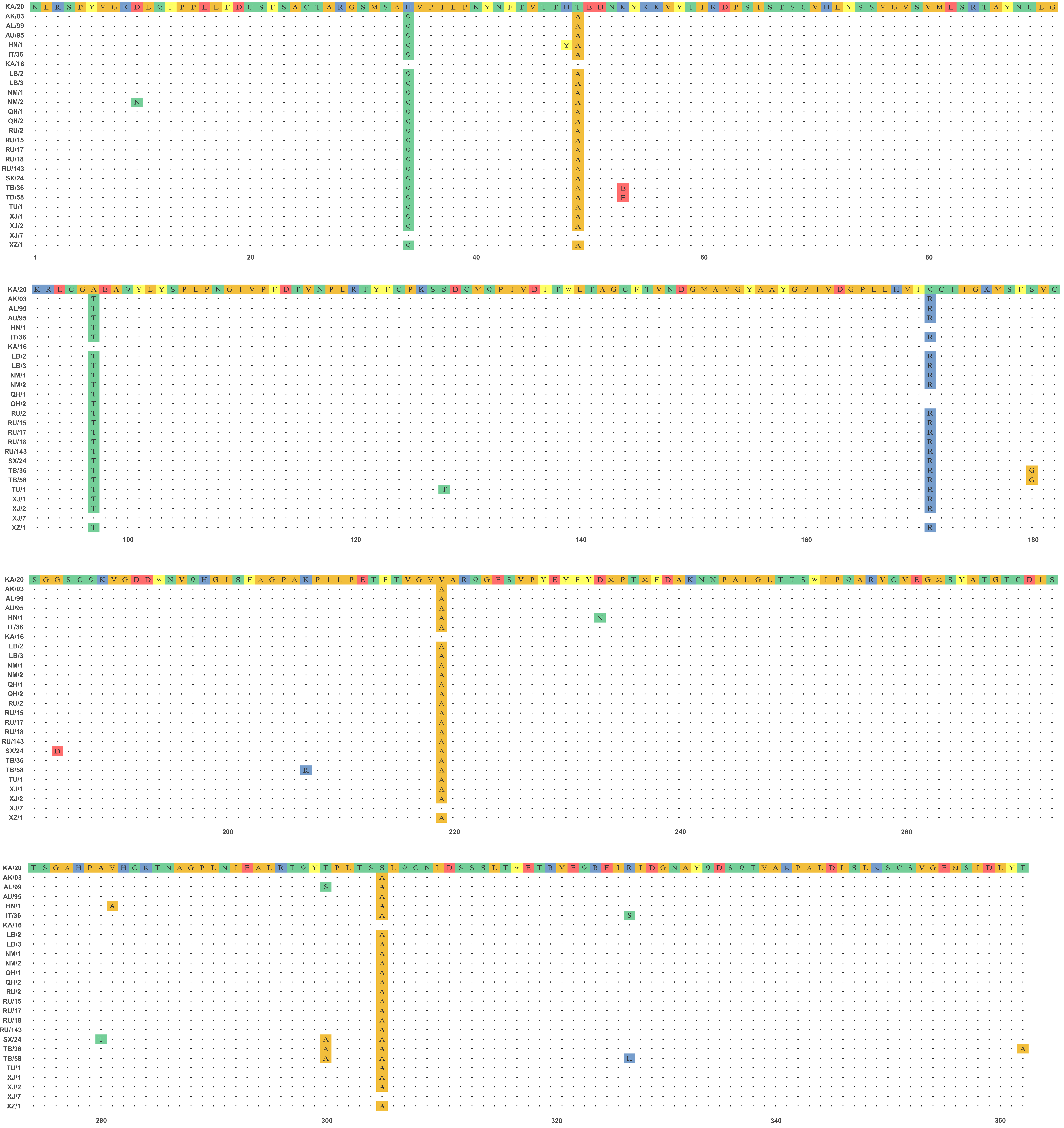
KA/20
AK/03
AL/99
AU/95
HN/1
IT/36
KA/16
LB/2
LB/3
NM/1
NM/2
QH/1
QH/2
RU/2
RU/15
RU/17
RU/18
RU/143
SX/24
TB/36
TB/58
TU/1
XJ/1
XJ/2
XJ/7
XZ/1
1
20
40
60
80
100
120
140
160
180
200
220
240
260
280
300
320
340
360
